# Development and validation of an indicator system for the modernization of rural public sports services in China

**DOI:** 10.3389/fpubh.2026.1941012

**Published:** 2026-09-10

**Authors:** Xueting Gao, Jincao Zhai

**Affiliations:** ^1^School of Physical Education, Ludong University, Yantai, Shandong, China; ^2^School of Physical Education and Sport Science, Qufu Normal University, Qufu, China

**Keywords:** community sport governance, indicator system development and validation, public health policy, public service modernization, rural health promotion, rural public sports services

## Abstract

**Background:**

Rural public sports services are increasingly important for promoting physical activity, reducing rural health inequities, and strengthening community-based public service governance. However, existing studies have mainly examined service supply, satisfaction, or policy narratives, and have paid less attention to how modernization can be measured as a systemic and cross-sectoral process. Objective: This study develops and empirically validates an indicator system for the modernization of rural public sports services in China.

**Methods:**

Drawing on structural functionalism, new public service theory, and systems science, the study conceptualizes modernization as a dynamic process linking resource input, service transformation, demand feedback, and institutional environment. A mixed-methods design was adopted, including literature review, semi-structured interviews, three rounds of Delphi consultation, a survey of 367 rural residents in Shandong Province, confirmatory factor analysis, and AHP-entropy combined weighting. The study included a pilot survey with 105 valid responses and a formal survey of 367 rural residents in Shandong Province.

**Results:**

The validated system contains four domains, twelve subdomains, and thirty-six observed indicators: Factor Upgrading, Structural Optimization, Demand Responsiveness, and Institutional Transformation. Reliability and CFA supported the measurement structure, with acceptable but improvable model fit. Normalized AHP-entropy weights indicated that diversified funding allocation, health promotion and sports resource utilization, villagers’ self-organized participation, effective policy implementation, and multi-actor service supply are key priorities in the indicator system.

**Conclusion:**

The indicator system provides a multidimensional and diagnostically useful framework for assessing rural public sports service modernization beyond facility coverage alone. It can help local governments identify bottlenecks in resource allocation, service conversion, demand feedback, and institutional support, thereby supporting more integrated, evidence-informed, and people-centered public health planning in rural communities.

## 1 Introduction

Public sports services are an important component of contemporary public service systems. They safeguard citizens’ rights to participate in sport, promote physical activity, improve health and well-being, and contribute to social equity (1–3). In rural communities, public sports services also perform broader social functions. They create public spaces for interaction, connect residents through shared activities, strengthen community identity, and support grassroots governance (4–7). As rural health promotion and public service equity have become increasingly important policy concerns, rural public sports services have moved from a marginal welfare issue to a cross-sectoral topic linking public health, sport policy, community development, and rural governance (8–10).

In China, rural public sports services have developed alongside national strategies related to Healthy China, rural revitalization, the construction of a leading sporting nation, and the improvement of the national fitness public service system. Rural fitness squares, sports parks, community sports events, social sports instructors, village-level sports associations, and public physical activity programs have become more visible in many villages (10–12). These developments have improved basic access to physical activity opportunities. However, the expansion of facilities and activities has not fully resolved the mismatch between rural residents’ evolving needs and existing service supply (13, 14).

Several practical problems remain. Some villages have facilities but lack operation and maintenance. Some organize activities but fail to form a stable service system. Some provide services but lack channels for resident feedback. In other cases, services are standardized in ways that do not match local age structures, cultural preferences, daily schedules, or health needs (13, 15–20). These problems suggest that rural public sports service modernization should not be understood simply as adding facilities, increasing investment, or organizing more activities. Rather, it is a systemic transformation involving resource allocation, service supply, Demand Responsiveness, governance coordination, and institutional support (21–23).

Previous studies have examined public sports service equalization, rural sport governance, national fitness public services, resident satisfaction, and service performance (14, 24–27). This literature provides an important foundation. Nevertheless, three gaps remain. First, many studies focus on supply status or performance outcomes, while fewer studies examine modernization as a process-oriented change from basic coverage to system upgrading. Second, existing indicator systems mainly evaluate service level, quality, performance, or satisfaction, but they rarely capture modernization-specific dimensions such as resource upgrading, service structure optimization, Demand Responsiveness, and Institutional Transformation (21, 28–31). Third, demand feedback is often treated as an outcome of service delivery rather than as a force that reshapes resource allocation and service improvement (32–34). The novelty of this study is therefore its shift from outcome-centered evaluation to a modernization-oriented system framework that traces how resources, structures, resident demand, and institutions jointly help assess modernization capacity.

To address these gaps, this study develops and empirically validates an indicator system for the modernization of rural public sports services in China. The article is guided by three research questions: which dimensions should be included in the indicator system, whether these dimensions form a reliable and valid measurement structure, and which indicators are relatively more important for assessing modernization. By answering these questions, the study provides a modernization-oriented measurement framework for public sports service governance, rural health promotion, and cross-sectoral public service planning.

## 2 Literature review and theoretical framework

### 2.1 Rural public sports services and modernization

Scholars have reached a broad consensus on the connotation of rural public sports services, generally defining them as a package of publicly accessible physical activity resources offered to rural residents to safeguard their sports rights and boost population health (1, 6, 9, 10, 35). Nevertheless, most existing studies confine this construct to tangible inputs such as sports venues, fitness programs and instructor services, while paying insufficient attention to institutional arrangements, citizen participation mechanisms and cultural adaptability embedded within grassroots rural contexts (13, 32, 36).

Regarding the modernization of rural public sports services, prior literature often reduces this concept to quantitative expansion of physical facilities or superficial efficiency improvements in administrative management (12, 17, 18, 28). Such narrow interpretations fail to capture the systematic, resident-centered and evolutionary nature of service transformation. They also overlook the feedback loops between resource input, service delivery and resident demand highlighted by systems theory and the new public service theory (21, 30, 37). Accordingly, building on prior scholarship and centering the people-oriented value of rural public sports services, this study incorporates supply structure, Demand Responsiveness, institutional safeguards and community co-production into its analytical scope to redefine the modernization of rural public sports services. Rural public sports services constitute a dynamic and systematic process in which governments and relevant social actors provide facilities, activities, professional guidance, organizational support, information services and institutional arrangements to protect rural residents’ right to sport participation and satisfy their physical activity and health needs (10, 32, 34). Its modernization is far more than mere facility expansion (38). Instead, it refers to the systematic reconstruction of rural public sports service systems centered on residents’ demands, rights and well-being. A modernized rural public sports service system ought to be equitable and accessible, demand-responsive, collaborative, inclusive, resilient and sustainable. It should move beyond externally driven, project-based supply models toward a service paradigm marked by endogenous participation and iterative improvement (13, 33, 39, 40).

### 2.2 Theoretical foundations

This study integrates structural functionalism, new public service theory, and systems science to explain modernization as a linked process of structure, public value, and adaptation. Taken together, the three perspectives provide the conceptual basis for defining the indicator dimensions and translating them into measurable items (30, 37, 41).

#### 2.2.1 Structural functionalism

Structural functionalism was systematically formulated by Talcott Parsons. Its core proposition holds that every social system consists of multiple interdependent subsystems, each fulfilling four indispensable functions: adaptation, goal attainment, integration, and pattern maintenance (41, 42). Sustained orderly operation of the whole system relies on coordinated matching and complementary division of labor among all constituent elements. Widely adopted in research on public service provision, grassroots governance and community service system construction, this theory provides a classic analytical paradigm for untangling internal element relationships within public services and identifying the structural conditions that shape system performance (22, 23, 43).

When applied to the modernization of rural public sports services, rural public sports services constitute a compound social subsystem with a complete operational logic. Five core structural units shape this system: resource endowments, supply actors, operational procedures, resident needs, and institutional norms, each undertaking distinct functional roles. Resource elements perform the adaptive function for the system, furnishing material and human capital prerequisites for service delivery (13, 17, 18, 44). Multi-stakeholder supply and governance structures deliver the integrative function, consolidating scattered resources into standardized, accessible public sports services. Resident demands serve the goal-attainment function, anchoring the core value orientation of rural sports modernization (13, 24). Various policies, cultural frameworks and talent systems realize pattern maintenance, safeguarding the long-term stable operation of the system (24, 45, 46).

The absence of any single structural element or functional fragmentation across units readily leads to practical dilemmas including idle resources, supply–demand mismatches and governance failures. Accordingly, the modernization of rural public sports services cannot rely solely on investment in isolated individual elements; coordinated functioning of all structural components is indispensable (30, 31, 47, 48).

#### 2.2.2 The new public service theory

The new public service theory reflects on and revises the inherent limitations of new public management, which prioritizes efficiency and government dominance. It argues that the core mission of public services lies in safeguarding public interests, upholding citizens’ principal status, and establishing channels for democratic participation (37). Residents are framed not as passive recipients of public services, but as co-producers, evaluators and shared beneficiaries of public governance (32, 34, 36, 49). Distinct from conventional supply-oriented research, this theory highlights the role of demand feedback, public participation and value co-creation in advancing public service upgrading, making it highly compatible with the governance characteristics of acquaintance societies and villager self-governance in rural grassroots contexts (33, 50).

For the modernization of rural public sports services, the ultimate objective lies in improving rural residents’ health well-being and satisfying their diversified physical activity demands, rather than merely completing hardware construction such as venues and fitness equipment (1, 6, 19). From the lens of the new public service theory, villagers’ service quality perception, demand expression, participatory decision-making and outcome feedback constitute an important feedback role that supports iterative upgrading of public sports services (13, 32, 34). Establishing regular demand response mechanisms and engaging villagers and grassroots sports associations in service planning, event organization and performance evaluation addresses prominent pain points including homogenized rural sports services, disconnection between supply and demand, and insufficient public participation (10, 12, 14, 51, 52).

#### 2.2.3 Systems science theory

Systems science centers on four core attributes of complex systems: holism, non-linearity, feedback mechanisms and dynamic evolution. It posits that complex systems do not develop linearly from a single factor; rather, their development emerges from cyclic interaction and coupled adaptation among multiple internal links (30, 31, 48, 53). Continuous feedback loops operate within the system, whereby downstream output outcomes exert reverse effects on upstream input links to drive sustained optimization and iteration of the whole system (54). Breaking static and fragmented analytical lenses, this theory is well-suited for examining rural public sports services that involve multi-stakeholder participation, abundant constituent elements and long-term evolutionary trajectories (22, 23).

The modernization of rural public sports services exhibits typical characteristics of complex systems. Inputs of human, financial and venue resources cannot directly translate into modernized service outcomes; they require transformation and processing through supply models and governance mechanisms (21, 30). After services are delivered, villagers generate physical activity experiences and demand assessments, which act as feedback signals to readjust resource allocation and service content (13, 24, 26). Meanwhile, external institutional environments including policies, cultural norms and technologies constrain and incentivize all internal links throughout the system, forming a closed-loop circulation (9, 45, 55).

### 2.3 Theoretical framework and dimensional deduction

Drawing on the complementary logic of the three aforementioned theories, this study constructs a closed-loop analytical framework of “resource input, service transformation, demand feedback, institutional environment” to reconstruct the operational chain underpinning rural public sports service modernization. The four links maintain cyclically coupled relationships (30, 31, 56). Resource input functions as the material starting point of the entire system; service transformation acts as the intermediate carrier through which resources are translated into tangible public sports services; demand feedback provides an endogenous signal for iterative system upgrading; and the institutional environment, functioning as external constraints and safeguards, runs through all stages of resource investment, service provision and demand response to continuously regulate systemic operation (21, 23, 39, 52, 57–59). Based on the functional positioning of each link within the framework, four domains are deduced layer by layer and further decomposed into subdomains, enabling complete translation from theoretical logic to measurable indicators.

Theoretical-to-dimensional mapping. The three theories jointly support the four dimensions through different but complementary logics, rather than a rigid one-to-one correspondence. Structural functionalism explains why the system requires adaptive inputs and coordinated structures; new public service theory explains why resident participation and public value must shape service modernization; systems science explains why resource input, service transformation, demand feedback, and institutional support should be understood as a coupled cycle. On this basis, the four dimensions were derived as analytically distinct yet interdependent components.

First, Factor Upgrading represents the resource conditions that enable rural public sports services to operate and develop. Human resources, financial resources, material facilities, and technological resources provide the basic conditions for service provision. This dimension therefore includes human resource organization, financial resource allocation, and material and technological foundation (13, 17, 18). It focuses on whether resources are adequate, appropriately allocated, and capable of supporting sustained service improvement (46, 60–62).

Second, Structural Optimization represents the organizational and operational arrangements through which resources are converted into public sports services. It includes service supply structure, governance operation structure, and comprehensive service effectiveness (22, 23). This dimension emphasizes the organization of multiple actors, the standardization of service processes, and the extent to which resource inputs are translated into accessible, health-promoting, and locally appropriate services (39, 63, 64).

Third, Demand Responsiveness represents the role of residents’ experiences, expressed needs, and participation in service improvement. It includes service quality perception, demand response level, and demand expression and participation (13, 32, 34). This dimension treats residents as users, evaluators, participants, and sources of feedback within the service system (24, 26).

Fourth, Institutional Transformation represents the institutional and environmental conditions that support the continuity of modernization. It includes policy-economic support, cultural-health communication, and technology and talent support (9, 45, 55, 65). This dimension emphasizes the role of policy implementation, incentive arrangements, cultural adaptation, technological application, and talent development in sustaining rural public sports services (62, 66–68).

**Figure 1:**
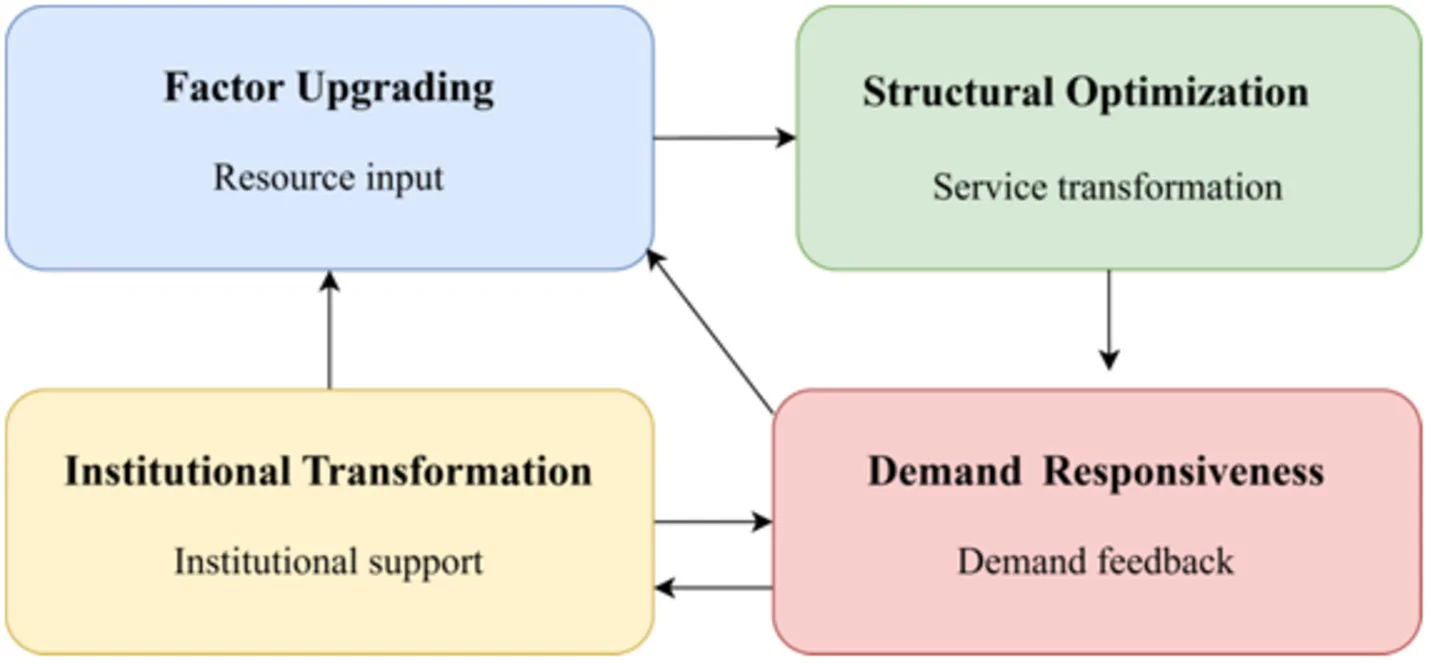
Analytical framework of rural public sports service modernization.

Overall, the four domains are not isolated or independent; they form interconnected and coupled relationships embedded within the closed-loop systemic framework. Inputs of factor resources are converted into public sports service outputs via supply structures. These outputs generate demand feedback among villagers, which, combined with external institutional environments, jointly adjust the scale and allocation mode of resource inputs in subsequent cycles (30, 31, 48) (see Figure 1).

## 3 Research design and methods

### 3.1 Overall research design

This study followed a sequential mixed-methods design comprising five linked stages: (1) theoretical deduction; (2) policy analysis and semi-structured interviews for item generation; (3) Delphi revision and content screening; (4) resident survey and CFA-based measurement assessment; and (5) AHP-entropy weighting. The sequence was designed to move from conceptual specification to context-sensitive item generation, expert refinement, empirical assessment, and indicator prioritization. The indicator system was described at three levels: four domains (Factor Upgrading, Structural Optimization, Demand Responsiveness, and Institutional Transformation), twelve subdomains, and 36 observed indicators. In the CFA stage, each domain was estimated as a separate nine-item within-domain measurement model, with its three subdomains represented by three observed indicators each. The analyses therefore provide evidence for the four within-domain measurement structures, but do not by themselves verify a pooled 36-item four-domain or higher-order model.

In the first stage, structural functionalism, new public service theory, systems science, and the relevant literature were used to establish the conceptual framework of rural public sports service modernization. Structural functionalism supports the adaptive, integrative, goal-oriented, and pattern-maintaining functions represented across the framework; new public service theory supports resident-centred Demand Responsiveness and co-production; and systems science explains the feedback and coupled relationships among the four domains. On this basis, four domains were identified: Factor Upgrading, Structural Optimization, Demand Responsiveness, and Institutional Transformation.

In the second stage, policy document analysis and semi-structured interviews were conducted to generate context-sensitive preliminary measurement items. The policy analysis covered major national policy documents, including the *Healthy China 2030 Outline, the National Fitness Plan* (2021–2025), and the Implementation Opinions on Promoting Sports to Boost Rural Revitalization. It also examined provincial and municipal national fitness implementation plans issued by Shandong Province and its subordinate prefecture-level cities. Statements related to the provision, administration, resource guarantee, participation, and evaluation of rural public sports services were extracted as semantic and contextual references for item development (69–71).

To complement the policy analysis, fieldwork was conducted in three prefecture-level cities and six counties or districts in Shandong Province. The sites were selected purposively to capture variation in rural development and public sports service conditions. Semi-structured interviews were conducted with 24 key informants: county education and sports officials (*n* = 3), township officials responsible for culture and sport (*n* = 4), village “two committees” members (*n* = 6), leaders of rural sports social organizations (*n* = 5), and actively exercising villagers (*n* = 6). Interviews lasted 45–90 min, with an average duration of 62 min. Recordings were transcribed verbatim and coded independently by three researchers using NVivo 12 Plus. Open coding yielded 187 initial concepts, axial coding yielded 43 categories, and selective coding yielded 14 core categories. Coding agreement exceeded Cohen’s kappa = 0.75; theoretical saturation was reached after the twentieth interview and confirmed through four additional interviews. The findings from the policy analysis and interviews were used to translate the theoretical domains into observable and operable measurement items.

In the third stage, Delphi expert consultation was used to assess and revise the preliminary item pool. Experts evaluated the importance, representativeness, clarity, operability, and dimensional attribution of each item. The final item-retention rule was mean > = 3.50, coefficient of variation (CV) < = 0.25, and item-level content validity index (I-CVI) > = 0.78. I-CVI was calculated as the number of experts assigning a relevance rating of 4 or 5 divided by the total number of experts (I-CVI = n_e/N). These quantitative criteria were applied together with written comments, theoretical attribution, conceptual distinctiveness, and measurability. Items that were theoretically important but marginally below one quantitative criterion were retained only after expert review and wording revision; items with weak relevance, conceptual duplication, or poor measurability were deleted, merged, or reassigned.

In the fourth stage, a resident questionnaire survey was conducted to assess the measurement structure. The survey data were used to examine item distributions, internal consistency reliability, common method bias, convergent validity, within-domain discriminant validity, and model fit through confirmatory factor analysis (CFA). The current analysis estimated four separate nine-item models rather than a pooled 36-item four-domain model or a higher-order model. Accordingly, the results support the proposed within-domain structures and should not be interpreted as direct evidence of cross-domain discriminant validity or complete four-domain structural validity.

In the fifth stage, the Analytic Hierarchy Process (AHP) and entropy method were combined to calculate the relative weights of the observed indicators. Experts constructed pairwise comparison matrices using Saaty’s 1–9 scale, and matrices were retained only when the consistency ratio (CR) was below 0.10. The entropy calculation treated all 36 indicators as positive indicators. For the resident response matrix *x*_ij_, min–max standardization was applied as $z_{\mathrm{ij}}=\frac{x_{\mathrm{ij}}-\min_{i}(x_{\mathrm{ij}})}{\max_{i}(x_{\mathrm{ij}})-\min_{i}(x_{\mathrm{ij}})}$ . Entropy was then calculated as $p_{\mathrm{ij}}=\frac{z_{\mathrm{ij}}}{\sum_{i=1}^{n}z_{\mathrm{ij}}}$ , $e_{j}=-\frac{1}{\text{In}(n)}\sum_{i=1}^{n}p_{\mathrm{ij}}\text{In}(p_{\mathrm{ij}})$ , and the entropy weight was $w_{j}^{E}=\frac{1-e_{j}}{\sum_{l=1}^{m}1-e_{l}}$ . AHP and entropy weights were combined with equal coefficients (0.50/0.50) because the study sought to balance normative expert judgment with observed resident-response variation. This equal-weight choice is a transparent baseline rather than a causal assumption; a baseline sensitivity analysis was conducted, but a broader sensitivity analysis (e.g., using a wider range of proportions such as 0.30/0.70 or performing sample perturbation) has not yet been conducted and should be added in future validation studies (69, 72–75).

**Figure 2:**
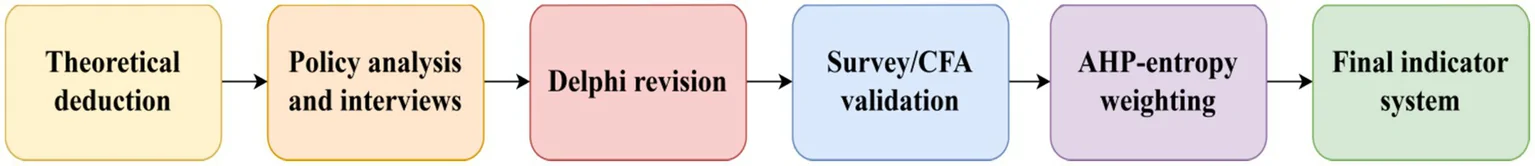
Sequential research procedure for scale development and empirical validation.

For the weighting audit, 17 pairwise-comparison matrices were specified across the hierarchy: one at the domain level, four at the subdomain level, and twelve at the observed-indicator level. The 20 experts’ ratings were used to define the pairwise comparisons, weights were obtained from normalized eigenvectors, and matrices were retained only when CR < 0.10. The first-level matrix had lambda_max = 4.206, CI = 0.0687, RI = 0.90, and CR = 0.0763. The complete matrix-level CR output should be retained as supplementary material so that the range and pass rate can be audited. For the entropy calculation, *i* = 1, *n* indexes respondents (*n* = 367) and *j* = 1, *m* indexes observed indicators (*m* = 36); all indicators were treated as positive indicators. When p_ij = 0, the term p_ij ln(p_ij) was defined as 0. The equal 0.50/0.50 combination was treated as a transparent baseline. Using the reported AHP and entropy vectors, sensitivity checks with AHP coefficients of 0.40 and 0.60 produced Spearman rank correlations of 0.982 and 0.990 with the baseline ranking; the highest-ranked funding-allocation and health/resource-utilization indicators remained among the leading priorities, although several middle ranks shifted modestly (see Figure 2).

### 3.2 Item generation through policy analysis and semi-structured interviews

Following the theoretical deduction, policy documents and semi-structured interviews were analyzed to translate the four conceptual dimensions into observable and context-sensitive measurement items. The policy analysis covered major national documents, including the Healthy China 2030 Outline, the National Fitness Plan (2021–2025), and the Implementation Opinions on Promoting Sports to Boost Rural Revitalization. Provincial and municipal national fitness implementation plans issued by Shandong Province and its subordinate prefecture-level cities were also reviewed. Statements concerning the provision, administration, resource guarantee, resident participation, and evaluation of rural public sports services were extracted as semantic and contextual references for item drafting (69–71).

Fieldwork was conducted in three prefecture-level cities and six counties or districts in Shandong Province. The sites were selected purposively to capture variation in rural development, public sports service provision, and governance conditions rather than to form a province-wide probability sample. Semi-structured interviews were conducted with 24 key informants: county education and sports officials (*n* = 3), township officials responsible for culture and sport (*n* = 4), village “two committees” members (*n* = 6), leaders of rural sports social organizations (*n* = 5), and actively exercising villagers (*n* = 6). Interviews lasted 45–90 min, with an average duration of 62 min. Recordings were transcribed verbatim and coded independently by three researchers using NVivo 12 Plus. Open coding yielded 187 initial concepts, axial coding yielded 43 categories, and selective coding yielded 14 core categories. Coding agreement exceeded Cohen’s kappa = 0.75; theoretical saturation was reached after the twentieth interview and confirmed through four additional interviews.

All interview recordings were transcribed verbatim after data collection. Open coding was used to identify statements related to the relevant subdomains. The coding process examined whether concrete practices corresponded to the conceptual meaning of each dimension, such as whether formal mechanisms for expressing residents’ demands functioned in practice, and whether grassroots practices revealed important local characteristics that were not sufficiently represented in the initial theoretical framework. Using the subdomains as the coding framework, at least three semantically representative excerpts from policy documents or interview transcripts were matched to each dimension. These excerpts served as contextual and semantic anchors for drafting the preliminary measurement items, which were then submitted to Delphi expert consultation for content evaluation and refinement.

### 3.3 Delphi expert consultation

The preliminary item pool generated through theoretical analysis, policy review, and semi-structured interviews was evaluated through Delphi expert consultation. Experts from universities, research institutes, sports authorities, and grassroots agencies, with backgrounds in sport science, public administration, sociology, or anthropology, assessed each item in terms of importance, representativeness, clarity, operability, and dimensional attribution (Table 1). A five-point scale was used, with 5 indicating very important and 1 indicating not important. The final item-retention rule was mean > = 3.50, CV < = 0.25, and I-CVI > = 0.78. These thresholds were used together with written comments, theoretical attribution, conceptual distinctiveness, and measurability; the first-round mean > 3.00 criterion was exploratory, and such items were reviewed further rather than automatically retained. Items that were theoretically important but marginally below one quantitative criterion were retained only after expert review and wording revision, whereas items with weak relevance, conceptual duplication, or poor measurability were deleted, merged, or reassigned (71–73).

**Table 1:** Characteristics of delphi experts.

Category	Group	*n*	%
Age	30–39	6	30.0%
Age	40–49	7	35.0%
Age	50 and above	7	35.0%
Education	Bachelor’s degree	5	25.0%
Education	Master’s degree	5	25.0%
Education	Doctoral degree	10	50.0%
Professional title	Intermediate	7	35.0%
Professional title	Associate senior	7	35.0%
Professional title	Senior	6	30.0%
Institution	University	9	45.0%
Institution	Sport administrative department	7	35.0%
Institution	Research institute	4	20.0%
Discipline	Sport science	9	45.0%
Discipline	Public administration	6	30.0%
Discipline	Sociology and related fields	5	25.0%

#### 3.3.1 First round: preliminary screening and structural optimization

In the first round, 22 questionnaires were distributed, 20 were returned, and all returned questionnaires were valid. Experts reviewed the initial pool of 64 tertiary items. After considering mean importance, CV, I-CVI, written comments, conceptual overlap, and measurability, the pool was reduced by 11 items on a net basis to 53 items; the reductions involved deletion, merger, and reassignment. The four domains were standardized as Factor Upgrading, Structural Optimization, Demand Responsiveness, and Institutional Transformation. Because the first round was exploratory, items with mean importance above 3.00 were retained for further review rather than automatically accepted.

#### 3.3.2 Second round: operationalization and hierarchical standardization

In the second round, the 20 experts who had validly participated in the first round received the revised questionnaire and a summary of the first-round revisions. All 20 questionnaires were returned and valid. The item pool was reduced by 17 items on a net basis, from 53 to 36 observed indicators. This round focused on operationalizing items so that they could be understood by rural residents and used in questionnaire measurement. Human resources were standardized as human resource organization, financial resources as financial resource allocation, and material and information resources were integrated into material and technological foundation. Demand response capacity was standardized as demand response level. Policy, economic, cultural, sports-technology, and talent factors were integrated into policy-economic support, cultural-health communication, and technology and talent support.

#### 3.3.3 Third round: stability confirmation and finalization

In the third round, the 20 experts received feedback from the first two rounds and the revised indicator system. The purpose was to confirm the stability of the hierarchical framework and obtain expert judgments for the subsequent AHP subjective weighting. The number of observed indicators remained 36; only minor wording adjustments were introduced to clarify the comprehensive level of sport-facility coverage and intelligent service capacity and to distinguish rural sport technology-service diffusion from intelligent sport-standard application.

#### 3.3.4 Expert authority and consensus

Expert participation, authority, and consensus were examined to assess the reliability of the Delphi process. In the first round, 22 questionnaires were distributed, 20 were returned, and all returned questionnaires were valid, yielding a response rate of 90.90% and a valid response rate of 100%. In the second and third rounds, 20 questionnaires were distributed and 20 valid questionnaires were returned in each round. Expert authority was calculated as the average of the judgment coefficient (Ca) and familiarity coefficient (Cs). Using the displayed Ca and Cs values in Table 2, the corresponding mean authority coefficients (Cr) are 0.854, 0.870, and 0.877 across the three rounds, all above 0.85. Kendall’s W increased from 0.292 to 0.353 and 0.367, and all values were statistically significant (*p* < 0.01), indicating that expert consensus improved across rounds and reached statistical significance. Because the retained item pool changed across rounds, W is interpreted primarily as within-round agreement and cross-round comparisons are made cautiously. Although the level of coordination was moderate rather than very high, the quantitative evidence and written comments together supported the stability of the final indicator system (72, 73).

**Table 2:** Expert authority coefficient and Kendall’s W coefficient of concordance.

Round	Rated items (k)	df = k − 1	Mean Ca	Mean Cs	Mean Cr	Kendall’s W	χ^2^	*p*-value
First round	64	63	0.842	0.865	0.854	0.292	367.920	<0.01
Second round	53	52	0.863	0.876	0.870	0.353	367.120	<0.01
Third round	36	35	0.875	0.879	0.877	0.367	256.900	<0.01

### 3.4 Survey and participants

The formal questionnaire survey was conducted among rural residents in communities across three prefecture-level cities and six counties or districts in Shandong Province. The sites were selected purposively to capture variation in rural development and public sports service conditions; recruitment was site-based and practical rather than probability-based. Within each selected village, investigators recruited eligible rural residents who were present during the field visit, could understand the questionnaire, and provided voluntary responses. Village organizations assisted with access and administration but did not complete responses on behalf of participants. A pilot survey was conducted in three administrative villages, yielding 105 valid responses. The pilot results showed an overall Cronbach’s alpha of 0.71 and a KMO value of 0.826. Trained investigators followed unified instructions for participant explanation, informed consent, on-site completion, and data checking. Of the 435 questionnaires distributed, 398 were returned; after excluding questionnaires with an obviously short completion time, more than five missing key items, more than eight consecutive identical responses, or obvious logical inconsistencies, 367 valid questionnaires remained, yielding an effective response rate of 84.37%.

Ethical safeguards were applied throughout the study. Participation in the interviews and questionnaire survey was voluntary and anonymous. Before data collection, participants were informed of the study purpose, the voluntary nature of participation, confidentiality protections, and their right to stop at any time; informed consent was obtained before interviews and questionnaire completion. No directly identifying information was collected, and the study involved minimal risk. The study was conducted in accordance with applicable principles for ethical social-science research, and all data were anonymized before analysis (see Table 3).

**Table 3:** Participant characteristics.

Variable	Category	*n*	%
Gender	Male	196	53.41%
Gender	Female	171	46.59%
Age	18 or below	4	1.09%
Age	19–29	12	3.27%
Age	30–39	32	8.72%
Age	40–49	64	17.44%
Age	50–59	130	35.42%
Age	60–69	90	24.52%
Age	70 or above	35	9.54%
Education	Primary school or below	77	20.98%
Education	Junior high school	171	46.59%
Education	High school or secondary vocational school	71	19.34%
Education	Junior college	34	9.26%
Education	Bachelor’s degree	12	3.27%
Education	Master’s degree or above	2	0.54%
Occupational identity	Traditional grain-farming farmer	167	45.50%
Occupational identity	Migrant worker farmer	74	20.16%
Occupational identity	Retired farmer	17	4.63%
Occupational identity	Social service farmer	29	7.90%
Occupational identity	Business-oriented farmer	22	5.99%
Occupational identity	Endogenous farmer	27	7.36%
Occupational identity	Exogenous farmer	1	0.27%
Occupational identity	Public institution employee	8	2.18%
Occupational identity	Other	22	5.99%
Health status	Very healthy	136	37.06%
Health status	Relatively healthy	120	32.70%
Health status	Fair	70	19.07%
Health status	Not very healthy	30	8.17%
Health status	Unhealthy	11	3.00%
Household annual income	Below 5,000 yuan	14	3.81%
Household annual income	5,000–9,999 yuan	18	4.90%
Household annual income	10,000–14,999 yuan	37	10.08%
Household annual income	15,000–19,999 yuan	46	12.53%
Household annual income	20,000–29,999 yuan	44	11.99%
Household annual income	30,000–49,999 yuan	65	17.71%
Household annual income	50,000–99,999 yuan	91	24.80%
Household annual income	100,000 yuan or above	52	14.17%

### 3.5 Data analysis

Descriptive statistics were conducted using SPSS 26.0 to examine the demographic structure of respondents and the distribution of the 36 observed variables. Reliability was assessed using Cronbach’s alpha and composite reliability (CR). Construct validity was examined through CFA using AMOS 28.0. The indicator system was described at three levels: four domains, twelve subdomains, and 36 observed indicators. The CFA stage estimated four separate nine-item within-domain measurement models, each containing three correlated subdomain constructs measured by three observed indicators. This design tested the within-domain measurement structure rather than a single pooled hierarchical model across all 36 indicators. Convergent validity was evaluated using standardized factor loadings, average variance extracted (AVE), and CR. Discriminant validity was evaluated using the Fornell–Larcker criterion only within each domain. A pooled four-domain model or higher-order model would be needed to establish cross-domain discriminant validity; such evidence is not claimed here. Common method bias was assessed through procedural controls and Harman’s single-factor test (76–80).

For the weighting analysis, expert consultation data were used for Delphi revision and AHP subjective weighting, whereas resident survey data were used for structural validation and entropy-based objective weighting. The AHP hierarchy comprised one domain-level matrix, four subdomain-level matrices, and twelve observed-indicator-level matrices. Experts used Saaty’s 1–9 scale; weights were derived from normalized eigenvectors, and each retained matrix met CR < 0.10. The first-level matrix had lambda_max = 4.206, CI = 0.0687, RI = 0.90, and CR = 0.0763. The entropy method used the 367 × 36 resident-response matrix, treated all indicators as positive, applied the equations shown in the preceding methods stage, and normalized the resulting entropy weights to 100% before combination. The combined weight was calculated as Wi = 0.5 × WAHP,i + 0.5 × WENT,i. The baseline sensitivity analysis using AHP coefficients of 0.40 and 0.60 yielded rank correlations of 0.982 and 0.990 with the 0.50 baseline, respectively. The complete matrix-level CR output and raw normalization file are archived as supplementary calculation material.

All numerical results were rechecked against the original data and calculation files before submission.

Table 4 presents four separate Fornell–Larcker panels, one for each within-domain measurement model. Each panel assesses discriminant validity only among the three subdomains estimated together in that model. Diagonal values are the square roots of AVE and off-diagonal values are inter-construct correlations. The results provide preliminary evidence of within-domain discriminant validity; they do not establish that the four domains are empirically distinct from one another.

**Table 4:** Within-domain discriminant validity.

Panel A. Factor Upgrading	HRO	FRA	MTF
HRO	0.846	0.264	0.298
FRA	0.264	0.924	0.492
MTF	0.298	0.492	0.859

Panel B. Structural Optimization	SSS	GOS	CSE
SSS	0.842	0.038	0.182
GOS	0.038	0.924	0.379
CSE	0.182	0.379	0.859

Panel C. Demand Responsiveness	SQP	DRL	DEP
SQP	0.918	0.595	0.379
DRL	0.595	0.907	0.342
DEP	0.379	0.342	0.867

Panel D. Institutional Transformation	PEG	CHC	TTS
PEG	0.849	0.482	0.308
CHC	0.482	0.924	0.368
TTS	0.308	0.368	0.859

Diagonal values were recalculated as the square roots of the corrected AVE values in Table 7; off-diagonal values are inter-construct correlations. Each panel evaluates the three subdomains within one domain. HRO, Human resource organization; FRA, Financial resource allocation; MTF, Material and technological foundation; SSS, Service supply structure; GOS, Governance operation structure; CSE, Comprehensive service effectiveness; SQP, Service quality perception; DRL, Demand response level; DEP, Demand expression and participation; PEG, Policy-economic support; CHC, Cultural-health communication; TTS, Technology and talent support. Cross-domain discriminant validity was not tested.

The AHP-entropy combined weighting method was used to estimate indicator priorities. AHP reflects expert judgment, policy orientation, and theoretical reasoning, whereas the entropy method reflects the dispersion and information contribution of resident survey data. The final combined weight was calculated by assigning equal weight to the two normalized vectors. These values represent relative diagnostic priorities and should not be interpreted as causal effects (74, 75).

### 3.6 Methodological rationale

The methodological design was intended to meet two requirements of indicator system development and validation research. The first requirement is content grounding. Because rural public sports service modernization is a context-sensitive construct, relying only on pre-existing scales from urban public service research would risk omitting rural characteristics such as village-level self-organization, local cultural adaptation, facility maintenance, and grassroots policy implementation. The use of literature analysis, policy review, and semi-structured interviews therefore ensured that the initial item pool reflected both theoretical concepts and local service realities (10, 13, 69).

The second requirement is empirical assessment. Indicators selected only through expert consultation may have content validity, but they do not necessarily form stable measurement structures among rural residents. For this reason, the Delphi procedure was followed by survey-based confirmatory factor analysis. This design allowed the study to assess whether the proposed indicators formed reliable within-domain measurement structures using resident-level data. Because a pooled 36-item four-domain or higher-order model was not estimated, the CFA evidence is interpreted as preliminary and domain-specific rather than as a complete test of the cross-domain hierarchy (74, 75, 77, 80).

## 4 Results

### 4.1 Final Indicator system

After literature analysis, interviews, and Delphi consultation, the final indicator system consisted of four domains, twelve subdomains, and thirty-six observed indicators. The structure is shown in Table 5.

**Table 5:** Indicator system for rural public sports service modernization.

Domain	Subdomain	Observed indicator
Factor upgrading	Human resource organization	Professional competence of rural sport personnel
Factor upgrading	Human resource organization	Scale suitability of rural social sport instructors
Factor upgrading	Human resource organization	Villagers’ participation in self-organized sport groups
Factor upgrading	Financial resource allocation	Rationality of diversified funding allocation for rural sport
Factor upgrading	Financial resource allocation	Efficiency of diversified funding supply for rural sport
Factor upgrading	Financial resource allocation	Market-oriented development capacity of rural sport service projects
Factor upgrading	Material and technological foundation	Integration of sport facility coverage and intelligent service capacity
Factor upgrading	Material and technological foundation	Suitability and maintenance level of sport facilities
Factor upgrading	Material and technological foundation	Coverage of sport information platforms and knowledge integration
Structural optimization	Service supply structure	Participation of multiple actors in rural public sports service supply
Structural optimization	Service supply structure	Innovation level of rural public sports service content
Structural optimization	Service supply structure	Capacity for transforming rural public sports service modes
Structural optimization	Governance operation structure	Clarity of responsibilities in rural public sports services
Structural optimization	Governance operation structure	Standardization of rural public sports service processes
Structural optimization	Governance operation structure	Risk prevention capacity of rural public sports services
Structural optimization	Comprehensive service effectiveness	Health promotion and sports resource utilization
Structural optimization	Comprehensive service effectiveness	Sport community connection and cultural adaptability
Structural optimization	Comprehensive service effectiveness	Synergistic economic and ecological benefits of rural sport
Demand responsiveness	Service quality perception	Perceived equity of rural public sports services
Demand responsiveness	Service quality perception	Perceived convenience of rural public sports services
Demand responsiveness	Service quality perception	Satisfaction with demand-service matching
Demand responsiveness	Demand response level	Timeliness of response to rural public sport needs
Demand responsiveness	Demand response level	Human-centered care in response to rural public sport needs
Demand responsiveness	Demand response level	Anticipatory capacity for upgraded rural public sport needs
Demand responsiveness	Demand expression and participation	Accessibility of channels for expressing rural public sport needs
Demand responsiveness	Demand expression and participation	Effectiveness of participation in rural public sport decision-making
Demand responsiveness	Demand expression and participation	Satisfaction with feedback processing for rural public sport needs
Institutional transformation	Policy-economic support	Effectiveness of rural revitalization sport policy implementation
Institutional transformation	Policy-economic support	Efficiency of grassroots sport autonomy and administrative approval
Institutional transformation	Policy-economic support	Improvement of sport funding incentives and market mechanisms
Institutional transformation	Cultural-health communication	Identification with traditional rural sport culture
Institutional transformation	Cultural-health communication	Inheritance and revitalization of traditional sport culture and intangible heritage
Institutional transformation	Cultural-health communication	Communication and penetration of fitness and health promotion
Institutional transformation	Technology and talent support	Improvement of rural sport talent systems
Institutional transformation	Technology and talent support	Innovation and diffusion of rural sport technology services
Institutional transformation	Technology and talent support	Development and application of intelligent standards for rural sport

The first dimension, Factor Upgrading, captures the input conditions that enable rural public sports services to operate. It includes human resource organization, financial resource allocation, and material and technological foundation. These indicators shift attention from counting resources to assessing whether resources are suitable, coordinated, and capable of supporting modernization (12, 17, 18).

The second dimension, Structural Optimization, captures the transformation process through which resources become service value. It covers service supply structure, governance operation structure, and comprehensive service effectiveness. This dimension is important because rural public sports services often fail not simply because resources are absent, but because existing resources are poorly organized or weakly connected to village life (22, 23, 64, 81).

The third dimension, Demand Responsiveness, captures the role of villagers’ needs in guiding service improvement. It includes service quality perception, demand response level, and demand expression and participation. The inclusion of this dimension distinguishes the present framework from conventional supply-side evaluation models. It treats residents’ demand not as a passive outcome but as an active source of feedback for service modernization (13, 24, 26).

The fourth dimension, Institutional Transformation, captures the broader environment that sustains long-term modernization. It includes policy-economic support, cultural-health communication, and technology and talent support. This dimension recognizes that rural public sports services require stable policy implementation, cultural legitimacy, health communication, and professional support to become sustainable (9, 45, 55).

### 4.2 Common method Bias test

Because the core questionnaire data were collected from the same respondents through self-report, common method bias was addressed through both procedural and statistical controls. Procedurally, the survey was administered anonymously, item wording was kept neutral and accessible, and respondents were informed that there were no right or wrong answers. Statistically, Harman’s single-factor test was conducted using unrotated exploratory factor analysis on all 36 measurement items. The results showed that nine factors had eigenvalues greater than 1, and the first factor accounted for 21.04% of the total variance. This proportion was below both the 50% threshold and the more conservative 40% criterion (76). Although Harman’s test is only a diagnostic procedure and cannot completely rule out common method bias, the result suggests that common method bias is unlikely to dominate the findings.

### 4.3 Reliability and validity testing

#### 4.3.1 Reliability testing

Cronbach’s alpha coefficients were calculated to examine the internal consistency of the scale. As shown in Table 6, the alpha coefficients of the four domains ranged from 0.802 to 0.847. The alpha coefficients of the twelve subdomains ranged from 0.806 to 0.940. All values exceeded the commonly recommended threshold of 0.70, indicating satisfactory internal consistency and measurement stability (78, 79).

**Table 6:** Reliability analysis of measurement indicators.

Domain	Cronbach’s alpha	Subdomain	Cronbach’s alpha
Factor upgrading	0.847	A1 Human resource organization	0.899
		A2 Financial resource allocation	0.884
		A3 Material and technological foundation	0.893
Structural optimization	0.806	B1 Service supply structure	0.877
		B2 Governance operation structure	0.940
		B3 Comprehensive service effectiveness	0.806
Demand responsiveness	0.819	C1 Service quality perception	0.934
		C2 Demand response level	0.887
		C3 Demand expression and participation	0.873
Institutional transformation	0.802	D1 Policy-economic support	0.882
		D2 Cultural-health communication	0.893
		D3 Technology and talent support	0.902

#### 4.3.2 Validity testing

#### 4.3.3 Convergent validity

As shown in Table 7, the 36 observed indicators are reported individually in the same code and item order used in Tables 5 and 8. The table provides the standardized loading, standard error, critical ratio, *p*-value, AVE, and CR for each item or subdomain. AVE and CR were recomputed from the standardized loadings shown in the table using the conventional formulas and rounded to three decimals. Standardized loadings ranged from 0.758 to 0.985. The first item within each subdomain was fixed for model identification; all freely estimated loadings were statistically significant at *p* < 0.001. AVE values ranged from 0.709 to 0.854 and CR values ranged from 0.879 to 0.946, supporting convergent validity and internal consistency at the subdomain level (77, 78).

**Table 7:** Convergent validity results.

Code	Observed indicator	Std. loading	S. E.	C. R.	*p*-value	AVE	CR
A1.1	Professional competence of rural sport personnel	0.758	Reference	Reference	Reference	0.715	0.882
A1.2	Scale suitability of rural social sport instructors	0.919	0.060	22.541	<0.001	0.715	0.882
A1.3	Villagers’ participation in self-organized sport groups	0.852	0.054	20.077	<0.001	0.715	0.882
A2.1	Rationality of diversified funding allocation for rural sport	0.843	0.081	22.403	<0.001	0.854	0.946
A2.2	Efficiency of diversified funding supply for rural sport	0.985	Reference	Reference	Reference	0.854	0.946
A2.3	Market-oriented development capacity of rural sport service projects	0.938	0.070	19.798	<0.001	0.854	0.946
A3.1	Integration of sport facility coverage and intelligent service capacity	0.796	Reference	Reference	Reference	0.738	0.894
A3.2	Suitability and maintenance level of sport facilities	0.949	0.029	22.338	<0.001	0.738	0.894
A3.3	Coverage of sport information platforms and knowledge integration	0.825	0.054	25.576	<0.001	0.738	0.894
B1.1	Participation of multiple actors in rural public sports service supply	0.758	Reference	Reference	Reference	0.709	0.879
B1.2	Innovation level of rural public sports service content	0.909	0.062	16.852	<0.001	0.709	0.879
B1.3	Capacity for transforming rural public sports service modes	0.852	0.060	16.808	<0.001	0.709	0.879
B2.1	Clarity of responsibilities in rural public sports services	0.843	Reference	Reference	Reference	0.854	0.946
B2.2	Standardization of rural public sports service processes	0.985	0.047	27.585	<0.001	0.854	0.946
B2.3	Risk prevention capacity of rural public sports services	0.938	0.056	25.905	<0.001	0.854	0.946
B3.1	Health promotion and sports resource utilization	0.796	Reference	Reference	Reference	0.738	0.894
B3.2	Sport community connection and cultural adaptability	0.949	0.070	19.647	<0.001	0.738	0.894
B3.3	Synergistic economic and ecological benefits of rural sport	0.825	0.085	17.965	<0.001	0.738	0.894
C1.1	Perceived equity of rural public sports services	0.930	Reference	Reference	Reference	0.843	0.941
C1.2	Perceived convenience of rural public sports services	0.964	0.028	28.186	<0.001	0.843	0.941
C1.3	Satisfaction with demand-service matching	0.857	0.031	28.597	<0.001	0.843	0.941
C2.1	Timeliness of response to rural public sport needs	0.917	Reference	Reference	Reference	0.822	0.933
C2.2	Human-centered care in response to rural public sport needs	0.902	0.059	17.752	<0.001	0.822	0.933
C2.3	Anticipatory capacity for upgraded rural public sport needs	0.901	0.072	20.963	<0.001	0.822	0.933
C3.1	Accessibility of channels for expressing rural public sport needs	0.836	Reference	Reference	Reference	0.751	0.900
C3.2	Effectiveness of participation in rural public sport decision-making	0.831	0.030	27.546	<0.001	0.751	0.900
C3.3	Satisfaction with feedback processing for rural public sport needs	0.929	0.033	16.378	<0.001	0.751	0.900
D1.1	Effectiveness of rural revitalization sport policy implementation	0.758	Reference	Reference	Reference	0.721	0.885
D1.2	Efficiency of grassroots sport autonomy and administrative approval	0.929	0.069	15.449	<0.001	0.721	0.885
D1.3	Improvement of sport funding incentives and market mechanisms	0.852	0.082	17.178	<0.001	0.721	0.885
D2.1	Identification with traditional rural sport culture	0.843	Reference	Reference	Reference	0.854	0.946
D2.2	Inheritance and revitalization of traditional sport culture and intangible heritage	0.985	0.065	21.088	<0.001	0.854	0.946
D2.3	Communication and penetration of fitness and health promotion	0.938	0.050	19.820	<0.001	0.854	0.946
D3.1	Improvement of rural sport talent systems	0.796	Reference	Reference	Reference	0.738	0.894
D3.2	Innovation and diffusion of rural sport technology services	0.949	0.067	21.673	<0.001	0.738	0.894
D3.3	Development and application of intelligent standards for rural sport	0.825	0.051	20.099	<0.001	0.738	0.894

**Table 8:** Normalized AHP subjective weights, entropy objective weights, and combined weights of the 36 observed indicators.

Observed indicator	AHP subjective weight	Entropy objective weight	Combined weight
Professional competence of rural sport personnel	2.999%	2.503%	2.751%
Scale suitability of rural social sport instructors	3.377%	1.446%	2.412%
Villagers’ participation in self-organized sport groups	3.826%	4.823%	4.325%
Rationality of diversified funding allocation for rural sport	4.646%	8.659%	6.652%
Efficiency of diversified funding supply for rural sport	4.970%	3.435%	4.202%
Market-oriented development capacity of rural sport service projects	3.851%	2.979%	3.415%
Integration of sport facility coverage and intelligent service capacity	3.867%	2.438%	3.153%
Suitability and maintenance level of sport facilities	3.921%	1.448%	2.684%
Coverage of sport information platforms and knowledge integration	2.895%	1.703%	2.299%
Participation of multiple actors in rural public sports service supply	2.096%	6.800%	4.448%
Innovation level of rural public sports service content	2.190%	2.342%	2.266%
Capacity for transforming rural public sports service modes	1.971%	1.552%	1.762%
Clarity of responsibilities in rural public sports services	2.566%	2.503%	2.534%
Standardization of rural public sports service processes	2.821%	2.041%	2.431%
Risk prevention capacity of rural public sports services	2.116%	2.503%	2.309%
Health promotion and sports resource utilization	3.212%	6.800%	5.006%
Sport community connection and cultural adaptability	2.577%	1.396%	1.987%
Synergistic economic and ecological benefits of rural sport	1.950%	2.868%	2.409%
Perceived equity of rural public sports services	2.549%	1.430%	1.990%
Perceived convenience of rural public sports services	2.573%	1.206%	1.890%
Satisfaction with demand-service matching	2.770%	3.435%	3.103%
Timeliness of response to rural public sport needs	2.924%	2.210%	2.567%
Human-centered care in response to rural public sport needs	2.625%	3.616%	3.120%
Anticipatory capacity for upgraded rural public sport needs	2.528%	2.556%	2.542%
Accessibility of channels for expressing rural public sport needs	2.302%	1.996%	2.149%
Effectiveness of participation in rural public sport decision-making	2.408%	1.446%	1.927%
Satisfaction with feedback processing for rural public sport needs	2.331%	2.438%	2.385%
Effectiveness of rural revitalization sport policy implementation	2.701%	6.166%	4.434%
Efficiency of grassroots sport autonomy and administrative approval	2.232%	1.985%	2.108%
Improvement of sport funding incentives and market mechanisms	2.632%	1.990%	2.311%
Identification with traditional rural sport culture	2.384%	1.953%	2.168%
Inheritance and revitalization of traditional sport culture and intangible heritage	2.439%	1.346%	1.893%
Communication and penetration of fitness and health promotion	2.087%	1.346%	1.717%
Improvement of rural sport talent systems	2.063%	2.438%	2.251%
Innovation and diffusion of rural sport technology services	2.409%	1.996%	2.203%
Development and application of intelligent standards for rural sport	2.183%	2.210%	2.197%
Total	100.000%	100.000%	100.000%

#### 4.3.4 Discriminant validity

Discriminant validity was evaluated using the Fornell–Larcker criterion. Because the CFA was conducted separately for the four domains, Table 4 reports four complete 3 × 3 matrices, one for each within-domain measurement model. Diagonal values represent the square roots of AVE, and off-diagonal values represent inter-construct correlations. In each panel, every diagonal value exceeded the corresponding within-domain correlations, providing preliminary evidence of discriminant validity among the three subdomains estimated together. This evidence does not establish cross-domain discriminant validity among the four domains (77, 80).

#### 4.3.5 Overall model fit

The fit indices for the four separate within-domain measurement models are presented in Table 9. Overall, the models demonstrated an acceptable but improvable level of fit. The CMIN/DF values ranged from 3.896 to 4.672, RMR from 0.032 to 0.062, GFI from 0.917 to 0.969, AGFI from 0.829 to 0.956, IFI and CFI from 0.947 to 0.976, TLI from 0.920 to 0.954, and RFI from 0.908 to 0.941. RMSEA ranged from 0.085 to 0.098. Although several indices were within or close to commonly used reference ranges, the relatively high RMSEA values and comparatively lower AGFI values in some models indicate that the fit is not optimal. The CFA results therefore provide general, rather than definitive, support for the within-domain structure of the indicator system (77, 82).

**Table 9:** Model fit indices for the four measurement models.

Model	CMIN/DF	RMR	RMSEA	GFI	AGFI	IFI	CFI	TLI	RFI	PNFI	PCFI	AIC (default/saturated)	Conclusion
Factor Upgrading	4.463	0.060	0.096	0.969	0.956	0.976	0.976	0.954	0.941	0.511	0.515	2743.869/136.793	Acceptable but improvable
Structural Optimization	3.896	0.062	0.085	0.917	0.843	0.947	0.947	0.920	0.908	0.626	0.631	2715.227/207.507	Acceptable but improvable
Demand Responsiveness	4.407	0.043	0.095	0.925	0.829	0.956	0.956	0.923	0.908	0.632	0.637	3004.937/195.777	Acceptable but improvable
Institutional Transformation	4.672	0.032	0.098	0.943	0.893	0.959	0.959	0.938	0.923	0.632	0.639	2203.349/154.134	Acceptable but improvable

For the Factor Upgrading model, the fit indices were CMIN/DF = 4.463, RMR = 0.060, RMSEA = 0.096, GFI = 0.969, AGFI = 0.956, IFI = 0.976, CFI = 0.976, TLI = 0.954, and RFI = 0.941. The incremental fit indices provided general support for the proposed measurement structure. However, the RMSEA value approached 0.10, indicating that the model fit was acceptable but improvable rather than uniformly good.

For the Structural Optimization model, the fit indices were CMIN/DF = 3.896, RMR = 0.062, RMSEA = 0.085, GFI = 0.917, AGFI = 0.843, IFI = 0.947, CFI = 0.947, TLI = 0.920, and RFI = 0.908. The incremental fit indices provided general support for the proposed structure, whereas the relatively lower AGFI indicated that the model fit was acceptable but improvable.

For the Demand Responsiveness model, the fit indices were CMIN/DF = 4.407, RMR = 0.043, RMSEA = 0.095, GFI = 0.925, AGFI = 0.829, IFI = 0.956, CFI = 0.956, TLI = 0.923, and RFI = 0.908. These results indicate an acceptable but improvable fit. In particular, the comparatively lower AGFI and the RMSEA value close to 0.10 warrant a cautious interpretation of the dimensional structure.

For the Institutional Transformation model, the fit indices were CMIN/DF = 4.672, RMR = 0.032, RMSEA = 0.098, GFI = 0.943, AGFI = 0.893, IFI = 0.959, CFI = 0.959, TLI = 0.938, and RFI = 0.923. Although the incremental indices provided general support for the proposed structure, the AGFI was below 0.90 and the RMSEA approached 0.10. The model fit should therefore be regarded as acceptable but improvable.

Taken together, the four separate CFA models provide preliminary empirical support for the proposed within-domain measurement structure. They do not by themselves establish cross-domain discriminant validity among Factor Upgrading, Structural Optimization, Demand Responsiveness, and Institutional Transformation. Future research should test a pooled 36-item four-domain model or a theoretically justified higher-order model using larger and independent samples, cross-validation, and alternative model specifications.

### 4.4 Combined weighting results

This study employed a combined AHP-entropy weighting approach to estimate the relative priorities of the 36 observed indicators. The AHP weights reflected expert judgment and hierarchical synthesis, whereas the entropy weights reflected the discriminatory information contained in the responses of 367 rural residents. Because the two methods generate weights through different procedures, both vectors were aligned at the same 36-indicator level and normalized to a common scale before combination. Table 8 therefore reports three comparable columns: AHP subjective weight, entropy objective weight, and combined weight. The combined weight was calculated using an equal-weight linear combination of the two normalized vectors. These values represent relative diagnostic priorities within the indicator system and should not be interpreted as causal effects (74, 75).

The results show that the rationality of diversified funding allocation for rural sport received the highest combined weight. Funding supply efficiency also ranked relatively high. These findings suggest that modernization depends not only on the total amount of financial investment but also on whether funding is allocated efficiently across facility maintenance, activity organization, professional guidance, digital services, and resident participation (3, 12, 17, 83). Accordingly, local governments should improve the stability and transparency of funding arrangements, protect necessary operational and maintenance expenditure, and establish monitoring mechanisms that assess whether financial inputs are converted into continuous and accessible services. This implication corresponds to the Factor Upgrading dimension and highlights the need to move from simple input expansion toward coordinated and sustainable resource management.

Health promotion and sports resource utilization also received a relatively high combined weight. This result indicates that the modernization of rural public sports services should be assessed according to how effectively existing resources support physical activity, health improvement, and regular participation, rather than by facility coverage alone (1, 6, 25). In policy practice, local governments should strengthen coordination between public sports services and rural health-promotion programs, improve the utilization of existing venues, and develop activities that reflect local age structures, cultural preferences, daily routines, and health needs. Regular assessment of facility use, activity participation, and health-oriented service outcomes can help identify underused resources and guide subsequent service adjustments. This finding is closely related to Structural Optimization because it emphasizes the transformation of resource inputs into usable and health-promoting services.

Villagers’ participation in self-organized sport groups was also among the relatively highly weighted indicators. This finding highlights the importance of locally embedded participation in rural public sports service modernization. Walking groups, dance teams, basketball teams, sports associations for older adults, and volunteer groups can help identify residents’ needs, organize regular activities, sustain participation, and connect formal public services with everyday community life (7, 84, 85). Therefore, policy implementation should provide supportive conditions for village-level self-organization, including accessible venues, small-scale activity funding, volunteer training, safety guidance, and simplified coordination procedures. Villagers should be regarded not only as recipients of services but also as participants in service planning, organization, and feedback. This implication reinforces the Demand Responsiveness dimension by demonstrating the practical value of resident participation in adapting services to local conditions.

The relatively high weight of policy implementation further indicates that the modernization of rural public sports services depends on the effective translation of policy objectives into village-level practice. The existence of policy documents alone does not guarantee service improvement. Their practical value depends on the clear assignment of responsibilities, timely delivery of resources, continuity of implementation, local adaptation, and effective monitoring. Policy evaluation should therefore examine not only whether policies have been formulated but also whether they are implemented consistently and produce observable improvements in service access, utilization, and resident participation. This finding is consistent with the Institutional Transformation dimension and underscores the importance of stable and accountable institutional support.

Finally, the relatively high weight of multi-actor participation in service supply suggests that government-led provision alone may not be sufficient to support modernization. Rural public sports services require coordinated participation by government departments, village collectives, social organizations, schools, enterprises, volunteers, and residents (22, 23, 32). Policy measures should clarify the responsibilities of different actors, establish mechanisms for resource sharing and collaborative planning, and provide appropriate incentives and accountability arrangements. Such arrangements can expand service capacity, improve local adaptability, and reduce dependence on a single administrative provider.

Taken together, the combined weighting results identify five closely connected policy priorities: diversified and efficient funding allocation, health-oriented resource utilization, support for villagers’ self-organization, effective policy implementation, and multi-actor service provision. These priorities correspond, respectively, to resource upgrading, service transformation, Demand Responsiveness, institutional support, and collaborative governance. The findings therefore suggest that rural public sports service modernization should be pursued as a coordinated system-improvement process rather than through isolated increases in facilities, funding, or activity numbers.

## 5 Discussion

### 5.1 From service evaluation to a systemic modernization framework

This study developed an indicator system for rural public sports service modernization and assessed its content, reliability, convergent validity, and within-domain discriminant validity. Beyond identifying important domains of service modernization, the framework provides a measurement tool that can help local governments move from siloed service reporting toward integrated public health planning across sport, health, education, and rural governance. The findings indicate that modernization is not adequately captured by facility coverage, activity frequency, or resident satisfaction alone. Instead, it should be understood as a multidimensional governance process in which resources, service structures, resident demand, and institutional support interact.

The framework’s novelty lies in its diagnostic orientation. Unlike evaluations centered mainly on service quality, performance, or satisfaction, it examines modernization as a system-oriented process linking resource conditions, service structures, resident demand, and institutional support. Its purpose is not only to assess whether services are satisfactory, but also to identify which part of the modernization process requires policy attention.

### 5.2 Demand feedback as a governance signal

The Demand Responsiveness dimension confirms the importance of residents’ perceptions, Demand Responsiveness, and participation in demand expression. In rural public sports services, demand feedback should not be viewed only as a final evaluation of service satisfaction. It is also a directional signal that guides service adjustment and system upgrading (13, 86–88). When villagers can express needs, participate in decisions, and see feedback processed, public sports services become more adaptive and more closely connected to everyday life.

The interview evidence also helps explain why demand feedback is a system-level feedback signal rather than merely an outcome variable. Grassroots respondents repeatedly pointed to mismatches between top-down facility provision and actual village-level activity needs. This finding supports the inclusion of demand expression channels, participatory decision-making, and feedback processing satisfaction as core indicators. For rural public sports services, demand feedback can inform self-organization, resource allocation, service timing, content, and maintenance decisions.

### 5.3 Resource input must be converted through structural optimization

The weighting results show that rational diversified funding allocation and efficient funding supply were among the highest-ranked indicators. This finding indicates that the key issue is not simply whether funding exists, but whether financial resources are allocated across facility maintenance, activity organization, professional guidance, digital services, and participation support in a balanced way. In practice, county and township governments should move from one-time infrastructure investment toward full-cycle budgeting that covers construction, operation, maintenance, personnel training, and service evaluation (17, 22, 23, 72).

### 5.4 Institutional transformation stabilizes long-term modernization

Institutional Transformation provides policy, economic, cultural, technological, and talent-related support for rural public sports service modernization. Rural public sports services are public, long-term, and community-based. They cannot rely solely on temporary projects or short-term campaigns. Stable policy implementation, funding incentives, cultural communication, technology diffusion, and talent systems are necessary to support sustainable service development (13, 45, 68).

### 5.5 International relevance

Although this study is based on rural China, the framework has relevance beyond the Chinese context (20, 89, 90). Many rural communities worldwide face similar challenges, including unequal access to physical activity opportunities, ageing populations, limited public resources, weak service responsiveness, and fragmented community governance. The framework of resource input, service transformation, demand feedback, and institutional environment offers a comparative tool for studying rural public sports services and community-based physical activity services in different contexts (8, 19, 91).

### 5.6 Theoretical contributions

This study contributes to theory in three ways. First, it reframes rural public sports service modernization as a dynamic process rather than a static service level. Previous studies have often measured service supply, service quality, or resident satisfaction (14, 92, 93). The present study identifies the interrelated dimensions relevant to modernization and organizes them into a functional system. This helps explain why similar resource inputs may be associated with different observed service conditions across villages.

Second, the study integrates public service governance and community sport perspectives. Public sports services are not only administrative products delivered by government agencies. They are also community-based services co-produced by local organizations, social actors, and residents (32–34, 36, 94). By including demand expression and participation and self-organized sport participation, the study highlights the role of citizens in rural public service modernization.

Third, the study extends the application of indicator system development and validation methods to rural public sports service research. The combination of qualitative item generation, Delphi consultation, CFA validation, and AHP-entropy weighting provides a replicable methodological route for measuring complex public service constructs in rural contexts (69, 72, 73, 77).

### 5.7 Measurement for change: from Siloed indicators to systemic public health planning

The value of this study is not limited to measuring the modernization of rural public sports services. More importantly, it shows how information generated from a multi-dimensional indicator system can be used to connect fragmented policy areas into a more coherent planning process. Rural sports services are closely linked with health promotion, community participation, education, village governance, and social organization, so their modernization cannot be understood as a single-sector issue.

From the M4C perspective, the four dimensions identified in this study can support a more system-oriented approach to public health planning. Factor Upgrading highlights the resource conditions that enable action; Structural Optimization shows how resources are converted into services; Demand Responsiveness captures the information flowing upward from residents; and Institutional Transformation reflects the broader enabling environment. Together, these dimensions help move measurement beyond isolated indicators toward a system that is dynamic, information-oriented, inclusive, interactive, and people-centered.

This means the indicator system can be used not only to assess village-level sports service performance, but also to support coordination among sport departments, health agencies, education actors, township governments, and village organizations. In this sense, the study contributes to the M4C goal of using information to improve decision-making and strengthen sustainable systems.

### 5.8 Practical significance

The practical value of the indicator system lies in its diagnostic use. Local governments can use the four dimensions to identify which part of the rural public sports service system constrains modernization. If Factor Upgrading is weak, the priority should be stable funding, professional personnel, facility maintenance, and digital access. If Structural Optimization is weak, the priority should be clearer responsibility allocation, multi-actor coordination, standardized service processes, and risk prevention. If Demand Responsiveness is weak, the priority should be routine demand collection, participatory decision-making, and transparent feedback processing. If Institutional Transformation is weak, the priority should be policy implementation, funding incentives, cultural-health communication, technology diffusion, and talent support. The high weights of health promotion and sports resource utilization also suggest that public sports services should be assessed by their actual contribution to physical activity participation, resource use, social connection, and everyday well-being. Therefore, the indicator system should not be used only as a one-time evaluation checklist. It can serve as a continuous learning tool that helps counties, townships, and villages compare changes over time, identify weak links, and coordinate sport, health, education, culture, and grassroots governance resources (21, 22, 39).

For villages with weak Factor Upgrading, policy should focus on professional guidance, funding allocation, facility maintenance, and digital platform construction (12, 61, 62, 95). For villages with weak Structural Optimization, policy should focus on multi-actor coordination, service content innovation, process standardization, and risk prevention (22, 23, 64). For villages with weak Demand Responsiveness, policy should establish demand expression channels, participatory decision-making mechanisms, and feedback processing systems (13, 34, 96). For villages with weak Institutional Transformation, policy should improve implementation accountability, cultural-health communication, technology diffusion, and talent systems.

## 6 Conclusion and policy implications

### 6.1 Main findings

This study set out to identify, assess, and prioritize the dimensions that underpin the modernization of rural public sports services. The findings indicate that modernization is not primarily a matter of expanding sports facilities or increasing administrative inputs. Rather, it depends on whether rural service systems can organize resources, convert them into effective and health-promoting services, receive and process residents’ demand signals, and sustain these processes through institutional support. In this sense, the indicator system transforms fragmented service information into diagnostic evidence for system learning and cross-sectoral public health planning.

The first contribution of the study is the development and preliminary measurement assessment of a four-domain framework. Factor Upgrading captures the resource base of modernization; Structural Optimization captures the capacity to convert resources into service effectiveness; Demand Responsiveness captures residents’ perceived quality, responsiveness, and participation; and Institutional Transformation captures the policy, cultural, technological, and talent conditions that stabilize long-term service improvement. The CFA results support the separability of the three subdomains within each domain, but cross-domain discriminant validity was not directly tested. The framework can therefore be used cautiously to diagnose different bottlenecks in village-level service modernization.

The second contribution lies in the weighting results. The highest-weighted indicators are not limited to traditional input variables. Diversified funding allocation, health promotion and sports resource utilization, villagers’ self-organized participation, effective policy implementation, and multi-actor service supply occupy prominent positions. This pattern shows that modernization requires a shift from resource possession to resource conversion, and from government-led delivery to demand-responsive co-production.

The third contribution concerns the role of resident demand. The results suggest that demand expression and self-organized participation should be treated as active sources of system intelligence rather than passive outcomes. For rural public sports services, residents are not only users who evaluate service quality after delivery; they are also actors who identify needs, sustain participation, and provide feedback for service adjustment. This finding is central to the policy relevance of the study and aligns with the Measurement for Change emphasis on using information to strengthen systems rather than to describe silos.

### 6.2 Policy implications

First, policy should move from facility-centered expansion to diagnostic and cross-sectoral governance. The indicator system can be used to identify which dimension is constraining a particular village and to align sport, health, education, cultural, social service, and rural governance resources accordingly. Villages with weak Factor Upgrading need stable funding, professional guidance, facility maintenance, and digital support. Villages with weak Structural Optimization need clearer responsibilities, stronger multi-actor coordination, and more effective service processes.

Second, local governments should institutionalize demand feedback as a routine part of service governance. Demand collection should not be limited to occasional satisfaction surveys. Villages should establish regular channels through resident meetings, digital platforms, activity evaluations, and public response procedures. The key is to ensure that expressed needs are recorded, classified, responded to, and translated into adjustments in service content, timing, facility maintenance, and activity organization. This will help shift rural public sports services from top-down provision to adaptive governance.

Third, policy should support resident co-production rather than treating villagers as passive beneficiaries. Self-organized groups, sport volunteers, sports associations for older adults, walking groups, square dance teams, and youth sport groups can help maintain participation and connect services with everyday rural life. Public support for these groups should include small grants, training opportunities, access to venues, safety guidance, and recognition within village governance systems.

Fourth, implementation should be differentiated across governance levels while maintaining system-wide coordination. County-level authorities should focus on policy coordination, financial guarantees, data platforms, and professional training. Township-level authorities should coordinate service resources across villages and monitor implementation quality. Village-level organizations should mobilize residents, maintain feedback channels, support self-organization, and adapt services to local demographic and cultural conditions. Such multi-level coordination can help transform fragmented local information into shared evidence for public health planning and service integration.

Finally, rural public sports services should be embedded in a broader cross-sectoral public health ecosystem that links sport, health promotion, cultural life, volunteer service, education, and grassroots governance. The policy goal is not simply to increase the number of activities, but to build an integrated community-based service system in which sport contributes to physical activity, social connection, health equity, and everyday well-being. This integrated approach is especially important for villages facing ageing populations, out-migration, and uneven access to health-promoting public resources. The indicator system should therefore be used as a routine monitoring and learning tool rather than as a one-time research product. Local governments can apply it periodically to identify weak links, compare changes across villages, and assess whether modernization is becoming more balanced, inclusive, and sustainable. Used in this way, the framework can support continuous system learning, cross-sectoral coordination, and evidence-informed public health planning, which are central to the Measurement for Change perspective.

## 7 Limitations and future research

This study has several limitations. First, the empirical survey data were collected from rural areas in Shandong Province. Although Shandong is an agricultural and populous province with a meaningful rural sport service context, the findings should be generalized cautiously to regions with different economic foundations, cultural traditions, demographic structures, and governance capacities. Future studies should use multi-province samples to test the regional adaptability of the indicator system. Second, the survey data used for indicator validation were cross-sectional and based on residents’ self-reported perceptions. Although reliability, validity, and common method bias were examined, future research should combine subjective survey data with objective administrative records, facility usage data, activity participation records, and health-related indicators. Third, the weighting results depend on both expert judgments and the dispersion of the survey data. The AHP-entropy approach improves balance between subjective and objective information, but the results may still be influenced by expert composition, judgment matrix design, sample characteristics, and the normalization procedure. Future research could compare AHP-entropy results with alternative weighting methods and conduct a broader sensitivity analysis (e.g., using a wider range of weight proportions such as 0.30/0.70 or performing sample perturbation). Fourth, this study validates the indicator system statistically, but it does not directly test whether higher indicator scores lead to long-term improvements in physical activity, health outcomes, or governance performance. Future studies should use longitudinal data, independent validation samples, system dynamics simulation, structural equation modeling, or configurational methods to examine how different combinations of factors produce rural public sports service modernization.

## Data Availability

The original contributions presented in the study are included in the article/supplementary material, further inquiries can be directed to the corresponding author/s.
