# Correction: Development and validation of an indicator system for the modernization of rural public sports services in China

**DOI:** 10.3389/fpubh.2026.1999977

**Published:** 2026-09-28

**Authors:** Xueting Gao, Jincao Zhai

**Affiliations:** 1 School of Physical Education, Ludong University, Yantai, Shandong, China; 2 School of Physical Education and Sport Science, Qufu Normal University, Qufu, China

**Keywords:** community sport governance, indicator system development and validation, public health policy, public service modernization, rural health promotion, rural public sports services

Affiliation School of Physical Education, Ludong University, Yantai, Shandong, China was omitted from the paper. This affiliation has now been added for author Xueting Gao.

The original version of this article has been updated.

